# Exact model reduction with delays: closed-form distributions and extensions to fully bi-directional monomolecular reactions

**DOI:** 10.1098/rsif.2014.0108

**Published:** 2014-06-06

**Authors:** Andre Leier, Manuel Barrio, Tatiana T. Marquez-Lago

**Affiliations:** 1 Okinawa Institute of Science and Technology, Okinawa, Japan; 2 Departamento de Informática, Universidad de Valladolid, Valladolid, Spain; 3 Integrative Systems Biology Unit, Okinawa Institute of Science and Technology, Okinawa, Japan

**Keywords:** chemical reaction networks, exact model reduction, distributed delays

## Abstract

In order to systematically understand the qualitative and quantitative behaviour of chemical reaction networks, scientists must derive and analyse associated mathematical models. However, biochemical systems are often very large, with reactions occurring at multiple time scales, as evidenced by signalling pathways and gene expression kinetics. Owing to the associated computational costs, it is then many times impractical, if not impossible, to solve or simulate these systems with an appropriate level of detail. By consequence, there is a growing interest in developing techniques for the simplification or reduction of complex biochemical systems. Here, we extend our recently presented methodology on exact reduction of linear chains of reactions with delay distributions in two ways. First, we report that it is now possible to deal with fully bi-directional monomolecular systems, including degradations, synthesis and generalized bypass reactions. Second, we provide all derivations of associated delays in analytical, closed form. Both advances have a major impact on further reducing computational costs, while still retaining full accuracy. Thus, we expect our new methodology to respond to current simulation needs in pharmaceutical, chemical and biological research.

## 1. Introduction

One of systems biology's main goals is to build predictive, quantitative models of biochemical processes, allowing for a better understanding of complex mechanisms in living cells. While the ultimate goal of understanding all simultaneous relevant mechanisms and their specific interactions is desirable, its realization is quite overwhelming, and in general simply unfeasible. This holds especially true when aiming for accurate predictions of reaction systems, such as gene expression and cell signalling pathways, owing to the size of the system and the intrinsic multi-scale nature of all underlying chemical processes. However, such limitations also apply to large-scale interaction models in chemistry and physics.

Therefore, researchers have become increasingly interested in developing techniques for the simplification or reduction of complex (bio)chemical systems. The desired result of such techniques is an equivalent model that includes much fewer elements but captures all essential dynamics, ultimately producing an equivalent behaviour of selected ‘species of interest’ (SOI). The latter refers to chemical/molecular species that answer specific biological/chemical questions, or those that may be observable by experimental techniques. Some of the known reduction techniques for chemical reactions systems involve lumping [1,2], sensitivity analysis [3] or time-scale analysis [4–6] (see also [7]). In these scenarios, all species that do not affect the output beyond a predefined threshold are removed, and the simulation of the system becomes an approximation of the exact solution.

Another complementary way to circumvent the so-called crux of dimensionality is to partition large systems into composite parts that can be dealt with independently. This is a logical and practical way to proceed, and in fact has been experimentally adopted in many synthetic biology applications (the so-called ‘plug and play’ models). By analogy, one tempting modelling strategy would be to combine both approaches, i.e. to partition a full system into parts that can be combined with the bonus of reducing each of those parts into much simpler models. Ideally, the latter would also result in much lower total computational costs.

Separately, over the past decade, it has been widely recognized that stochastic phenomena play an essential role in many biochemical processes. This is particularly notable in the small-scale cellular machinery, where certain key molecules occur in low numbers. Fluctuations in such numbers are often statistically significant and relevant to the overall system dynamics. For instance, intrinsic noise has been reported to have an impact on cellular gene expression and regulation [8], cellular differentiation [9], (ion) channel gating in neurons [10], pattern formation [11] and evolution [12]. As a consequence, modelling and simulation frameworks that are able to represent stochastic systems accurately have become increasingly popular. So, if one is to reduce a system exactly, then its stochastic nature should also be taken into consideration.

The widely accepted formalism for describing stochastic reaction systems in well-mixed scenarios is the so-called chemical master equation (CME), a system of ordinary differential equations (ODEs) describing the time evolution of the probabilities for observing the system in each possible state. In certain cases, the CME can be solved analytically. However, such analytic solutions are limited to specific systems [13,14]. Alternatively, finite state projections of generalized systems can be obtained [15,16] that effectively reduce the state space, or one may simply resort to running a stochastic simulation algorithm (SSA) to obtain trajectories representing the time evolution of the numbers of molecules within a simulation volume [17–19]. The latter is the easiest option of all, if one is solely interested in the time evolution of molecular species, but the main drawback of using an SSA is that it can become inefficient for systems with large numbers of molecules and/or reactions.

Recently, we reported a new methodology for reducing certain types of reaction systems [20], where a time delay substituted whole sets of chemical reactions, yielding an *exact* solution for the user-defined SOI. The rationale behind this reduction was the following: sets of chemical reactions do not produce fully functional products instantaneously. Additionally, reactions are coupled, in the sense that many reaction products can be the reactants of other reactions in the system of interest. These combined effects, under specific circumstances, can be encapsulated in one or more delayed reactions. As we showed, such delayed reactions can be in turn simulated with a delay SSA (DSSA), the extension of the classical SSA to systems with delays. If the lumping is correctly done, the result is a lower dimensional system that retains all essential behaviour, without any loss of accuracy in the dynamics of the SOIs. Namely, the dynamics of the SOIs in the original and the reduced model are identical. To be more precise, the solution of the delay chemical master equation (DCME) associated with the abridged model is equal to the solution of the CME for the original model with respect to the time evolution of the probability density function (PDF) of the state space involving only SOIs. When applied to the reduced model, the DSSA will then generate a trajectory that is sampled from the exact solution of the DCME—just like the SSA produces trajectories that are sampled from the exact solution of the underlying CME. This is a significant improvement over many acceleration techniques, such as tau-leap methods [21–23], which are only approximations of a true solution. Also, our reduction methodology is not limited to particular time scales, or separation of time scales to that extent. It can be applied irrespective of scales of kinetic parameters, and their scaling against each other.

Our approach in Barrio *et al*. [20] was based on the idea of random walks and first-arrival times. There, we imposed some restrictions on the kind of reaction blocks that could be lumped. These restrictions were related not only to the type of reactions contained in the blocks but also to how blocks could be connected among themselves. Namely, a linear chain of reactions composing a block
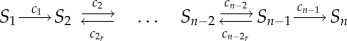
was shown to be exactly reducible to a single delayed reaction
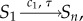
with appropriate delay distribution. However, exactness in the dynamics of the SOIs, here *S*_1_ and *S_n_*, required the irreversibility of the first and last reaction of the linear chain. Strategies to deal with certain types of bypass, degradation and synthesis reactions in the system were also provided.

In this paper, we extend our methodology and provide all derivations of associated delays in analytical, closed form. Quite naturally, this has a major impact on the efficiency of the simulation algorithm, as we use a DSSA procedure [24,25] and the overall efficiency of this type of algorithm depends greatly on the random number generation. Specifically, the efficiency and accuracy of our method increases considerably by readily knowing the expression of all associated delay distributions. However, there are many additional benefits to adopting our modified method, and the organization of this paper follows each extension systematically.

First, we consider fully reversible delay reaction blocks, aiming to reduce more general biochemical networks exactly. In this case, our abridgement is achieved by introducing delayed reactions for first-return times, whose PDFs can be calculated in a similar way to those of first-arrival times. Here, the time that a random walker needs to return to its initial state will be generally denoted as ‘first-return’, while the time that a random walker needs to arrive at another state will be generally referred to as ‘first-arrival’ (in the case of two or more SOIs). However, these definitions underlie some differences in specific cases, the subtleties of which will be formally introduced in §2. Analytic expressions for the first-arrival and first-return time distributions and for the probabilities of first-arrival versus first-return events are then derived. As we show, this also yields previously unknown analytic expressions of first-arrival distributions and reaction probabilities for molecules in systems with degradation reactions.

Second, in the past we had shown that PDFs of first-arrival times could be derived in closed form in the presence of backward bypass reactions. However, it did not seem possible for forward bypass reactions
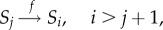
and these had to necessarily be dealt with numerically, by calculating matrix exponentials. Here, we demonstrate that delay distributions can be obtained in closed form also in the presence of forward bypass reactions. Importantly, this holds true for systems with or without simultaneous backward bypass reactions. This not only provides modellers with a more straightforward approach, but also dealing with forward bypass reactions in closed form could further reduce total computational costs, which was the motivation behind our model reduction in the first place.

Third, we provide closed form expressions for degradation and synthesis reactions in the system. We also provide separate expressions for systems in which all of the above reactions take place. The latter increases the applicability of our method to open systems.

Lastly, we discuss our results and benchmark the proposed method in terms of both efficiency and accuracy. All proofs and mathematical subtleties of the method can be found in the electronic supplementary material.

## 2. Preliminary definitions

**Figure 1.**
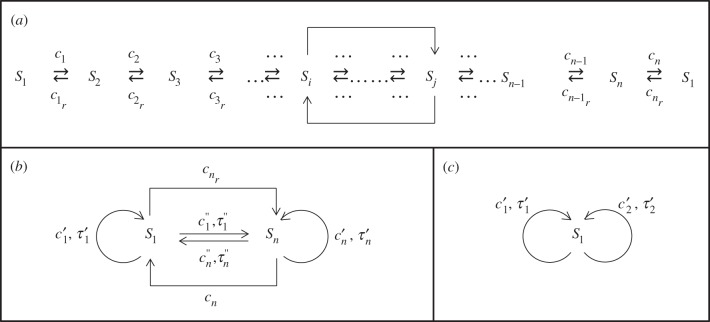
(*a*) Full reaction scheme including forward and backward bypass reactions (reactions between two species *S_i_* and *S_j_* for | *j – i*| > 1). (*b*) Abridged model for two SOIs, *S*_1_ and *S_n_*. This abridgement works, as long as *S*_1_ and *S_n_* are not part of any forward and/or backward bypass reaction. Loops refer to delayed reactions with first-return distributions. Arrows refer to either non-delayed reactions or delayed reactions with associated first-arrival distributions. Rates are denoted with *c* and delays with *τ*. The reactions with rates 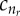 and *c_n_* are identical to those in (*a*). (*c*) Abridged model for one SOI, *S*_1_. This abridgement requires two delayed reactions with first-return time distributions, one for each outgoing reaction with *S*_1_ as the reactant in the original model. Note that rates and delays in (*c*) are different from those in abridgement scheme (*b*).

Abridgements of a linear bi-directional chain of reactions 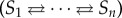 including closed circles 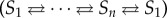 and forward (*S_j_* → *S_i_*, *i* > *j* + 1) and backward (*S_j_* ← *S_i_*, *i* > *j* + 1) bypass reactions can be done in several ways. The appropriate choice depends on the system and the SOIs. A representative system is illustrated in figure 1*a*, whereas two possible abridgements are illustrated in figure 1*b*,*c*.

The first abridgement scheme shown in figure 1*b* contains two SOIs, namely *S*_1_ and *S_n_*. We can explain the scheme in the context of a random walker that moves between positions, each of which is uniquely attributed to a species *S*_*i*_ in the system. Let us assume that a walker starts at position *S*_1_ and moves to position *S*_2_. Then, there is a non-zero probability that the walker returns to its original position *S*_1_, before arriving at the position of the other SOI, *S_n_*. Likewise, there is also a non-zero probability that the walker arrives at the position of the SOI *S_n_* without having revisited *S*_1_. Similar considerations hold for a walker that starts in position *S_n_* and moves to *S_n_*_−__1_. We will refer to the first scenario as ‘first-return’ and to the second scenario as ‘first-arrival’. Also, distributions describing the time that a walker takes to return to its starting position (without having visited any other SOI) will be called first-return distributions. Distributions describing the time that a walker takes to arrive at the position of another SOI (without having revisited its original position) will be called first-arrival distributions. Then, for each of these scenarios, first-returns and first-arrivals, we will define a reaction with associated delay distribution that lumps the walker's random movements between SOIs.

First-return reactions are symbolized as ‘loops’. Such reactions are consuming, delayed reactions *S_i_* → *S_i_*. Here, ‘consuming’ specifically denotes that the reactant is removed from the system state at the time that the reaction is triggered [24]. Likewise, delayed reactions *S_i_* → *S_j_*, representing first-arrival scenarios, are consuming reactions. For the given example, we obtain in total four delayed reactions, two with first-return and two with first-arrival distributions. Additionally, the two non-delayed reactions between the SOIs in the original model are taken over into the abridged model. Then, as we explain in more detail later, the reaction rates of the delayed first-return and first-arrival reactions will be the products of the rates of corresponding outgoing reactions in the original model (here *c*_1_ and 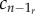) and the associated first-return and first-arrival probabilities, respectively.

It should be noted that, if we were only interested in one SOI, e.g. *S*_1_, then we do not need to ensure that all other species match the dynamics of the full model as well. This corresponds to the second abridgement scheme, illustrated in figure 1*c*. In this case, all delays correspond to first-return distributions and the associated reaction rates are the individual rates of outgoing reactions *S*_1_ → *X*, where *X* denotes a species within the original system (here, 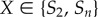).

For obvious reasons, any abridgement scheme depends on the network structure of the original system, the choice of SOIs and their ‘position’ within the reaction network. A simple recipe for generating such abridgement schemes is to create nodes for each SOI and directed links between any such nodes, i.e. reaction arrows, if the original model contains a sequence of reactions, where the second SOI can be produced from the first. Additional directed links need to be added for every other such sequence starting with a different outgoing reaction. In the abridged scheme, loops (also referred to as self-edges) represent delayed reactions that have the same species as both a reactant and product, and their delay is described by a first-return time distribution. Links between different SOIs represent delayed reactions with an associated first-arrival distribution.

In the following sections, we describe how to derive such delay distributions and rates of associated delayed reactions, for various types of reaction systems. In a first step, we attempt to create an abridged model of the system 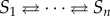 for the SOIs *S*_1_ and *S_n_*. Note that this system is identical to the full model in figure 1*a* minus the two reactions 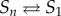 with rates 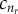 and *c_n_*. For that purpose, we need the following probabilities and delay distributions:

(i) *p_r_*_,1_ and *p_r_*_,*n*_: we define *p_r_*_,1_ as the probability of a walker starting at *S*_2_ to return to *S*_1_ without visiting *S_n_*; similarly, *p_r_*_,*n*_ is the probability of a walker starting at *S*_*n* −1_ to return to *S_n_* without visiting *S*_1_. Then, *p_a_*_,*n*_ = 1 − *p_r_*_,1_ and *p_a_*_,1_ = 1 − *p_r_*_,*n*_ are the probabilities of first-arrival at *S_n_* when starting at *S*_2_ and first-arrival at *S*_1_ when starting at *S*_*n* −1_, respectively.
(ii) *F_a_*_,*n*_ and *F_a_*_,1_: these are the cumulative distribution functions (CDFs) of first-arrival times of a random walker starting at *S*_2_ and arriving at *S_n_* and a random walker starting at *S*_*n* −1_ and arriving at *S*_1_, respectively.
(iii) *F_r_*_,1_ and *F_r_*_,*n*_: these are the CDFs of first-return times of a random walker starting at *S*_2_ and returning to *S*_1_ and of a random walker starting at *S*_*n* −1_ and returning to *S_n_*, respectively.

The probabilities *p_r_*_,1_ (*p_r_*_,*n*_) or *p_a_*_,*n*_ (*p_a_*_,1_) are then used as rate-adjusting factors in the DSSA to decide which of the two delayed reactions for leaving states *S*_1_ (*S_n_*) will be chosen: either the first-return reaction with rate *p_r_*_,1_*c*_1_ (*p_r_*_,*n*_*c*_*n* −1,*r*_) and delay distribution *F_r_*_,1_ (*F_r_*_,*n*_) or the first-arrival reaction with rate *p_a_*_,*n*_*c*_1_ (*p_a_*_,1_*c*_*n* −1,*r*_) and delay distribution *F_a_*_,*n*_ (*F_a_*_,1_). The choice of rates for delayed reactions becomes clear when comparing the abridged and original models. For illustration purposes, let us still use the system 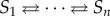 for the SOIs *S*_1_ and *S_n_*. Here, for example, a molecule of species *S*_1_ turns into a molecule of species *S*_2_ with rate *c*_1_. Now, to match the dynamics of *S*_1_ in the abridged system, one must define necessary outgoing reactions *S*_1_ → *X*, and the sum of all reaction rates associated with these outgoing reactions must equal *c*_1_. Thus, in our example, the rate *c*_1_ would have to be weighted over the two reactions *S*_1_ → *S*_1_ and *S*_1_ → *S_n_*, in accordance with the probabilities of observing a first-return, *p_r_*_,1_, or first-arrival event, *p_a_*_,*n*_ = 1 − *p_r_*_,1_, respectively. This leads to the appropriate rates.

## 3. First-arrival distributions in a fully reversible reaction chain

Denote 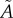 to be the *n* × *n* transition matrix of the reaction system3.1
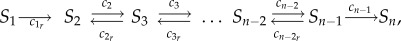
where *S*_1_ and *S_n_* can be interpreted as two absorbing boundaries for a random walker starting either at *S*_2_ or at *S*_*n* −1_. Moreover, let *p*_2_ = [0 1 0 … 0]^T^ and *p*_*n* −1_ = [0 … 0 1 0]^T^ be column vectors of length *n* and denote [*ν*]*_k_* the *k*th entry of a vector *ν*.

Then, 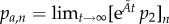 and 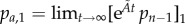 and
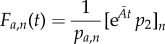

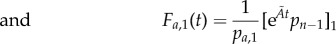
are the CDFs of first-arrival times at *S_n_* and *S*_1_, respectively (see also Methods section of Barrio *et al*. [20], III.F: Numerical solution of 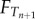).

Here, the sampling of *t* has to be done such that the CDFs are smooth and errors due to interpolation between time points become negligibly small. Thus, in order to obtain a close approximation of *p_a_*_,*n*_ and *p_a_*_,1_, *t* has to be very large since we are asking for the probability of the walker to eventually return to where it started.

In the study of Barrio *et al*. [20], we derived an analytic expression for the PDF of first-arrival times at *S_n_*. However, owing to the additional absorbing state in *S*_1_, our previous derivation breaks down when applied to the present scenario. Nonetheless, by using the exponential form above, one can still derive an analytic expression for the corresponding PDF of *F_a_*_,*n*_. Namely,
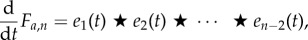
where 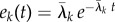 and 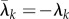 for all eigenvalues *λ_k_*(*k* = 1, … , *n* − 2) of 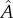 , which is the (*n* − 2) × (*n* − 2) rate matrix of the system3.2
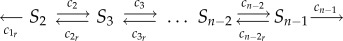
(see the electronic supplementary material, §S1, for a detailed proof). In other words, the PDF of the first-arrival-at-*S_n_* distribution is the convolution of exponential distributions with parameters 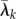, the absolute values of the eigenvalues of 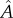. Its corresponding CDF is then given as3.3
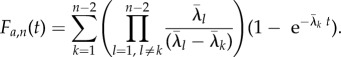
In addition, our derivation also yields an analytic expression for the probability of arrival at *S_n_*3.4
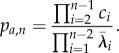


Conveniently, it can also be shown (see the electronic supplementary material, §S2) that *F_a_*_,*n*_(*t*) = *F_a_*_,1_(*t*) and3.5
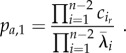


The fact that both first-arrival time distributions are identical is rather surprising and counterintuitive, in particular for systems where each reaction *S_i_* → *S_i_*_+__1_ is faster than its reverse reaction *S_i_*_+__1_ → *S_i_*. However, as it turns out, the sets of backward and forward reaction rates, 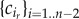 and 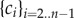, produce two effects: they (i) independently change the arrival probabilities and (ii) together determine the first-arrival distribution.

It is important to note that the obtained form of the first-return time distribution is basically identical to our previous result reported in the study of Barrio *et al*. [20]. However, its entire derivation had to be done from scratch, all over again, in order to show it can cope with the fully bi-directional reaction scheme. Also, it is worth emphasizing that the obtained expressions allow us to calculate the arrival probabilities and first-arrival CDFs much faster than by computing the matrix exponentials numerically. Lastly, the first-arrival distributions are no longer guaranteed to be identical once bypass reactions are introduced. This will be discussed in detail later (§5).

## 4. First-return distributions in a fully reversible reaction chain

Analogous to first-arrival distributions, we can obtain first-return distributions by calculating the matrix exponentials 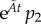 and 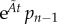 for various times *t*. More precisely, with 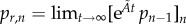 and 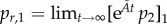 we obtain the CDFs of first-return to *S_n_* and *S*_1_ as
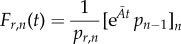

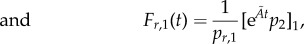
respectively. As mentioned before, the sampling of *t* has to be done such that the CDFs are smooth and errors owing to interpolation between time points become negligibly small.

We can then derive an analytic expression for *F_r_*_,1_(*t*), namely4.1
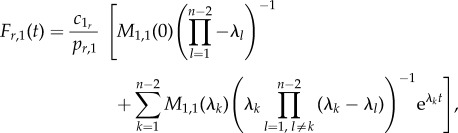
where *M*_1,1_(*s*) is the (1,1)-minor of 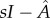 (see the electronic supplementary material, §S3). For a tridiagonal matrix 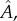 these minors can be calculated as 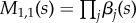 with
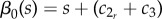

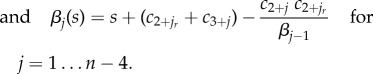
 Following the same steps, we obtain an equivalent analytic expression for *F_r_*_,*n*_(*t*)4.2
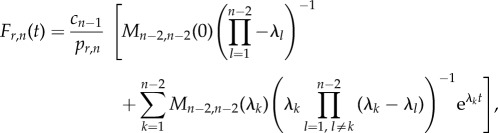
where *M_n_*_−2,*n*−2_(*s*) is the (*n* − 2, *n* − 2)-minor of 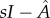. For a tridiagonal matrix 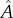, these minors can be calculated as described in the electronic supplementary material, §S3, yielding 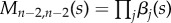 with
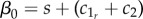

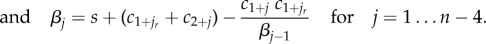
When taking the time limits of equations (4.1) and (4.2) to infinity, the left-hand side of both equations becomes 1 (owing to each being a CDF), while the second term in the sum on the right-hand side vanishes. By multiplying both sides of the resulting equations with the respective return probability, we obtain
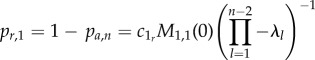

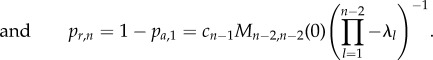


It should be noted that, while the two first-arrival distributions for chains of fully reversible reactions without bypass reactions are always identical (see below), the two first-return distributions are in general not identical.

## 5. Fully reversible reaction chain with additional bypass reactions

For the system studied in Barrio *et al*. [20], we had argued that the first-arrival distribution could also be derived analytically when additional backward bypass reactions
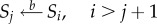
were present. In such a scenario, the corresponding rate matrix has additional non-zero entries above the super-diagonal, which does not invalidate the approach pursued in [20]. However, we expected all eigenvalues of the corresponding rate matrix to be real, which is not necessarily the case. Eigenvalues are either real or come in pairs of complex conjugates. As it turns out (see the electronic supplementary material, §S4), pairs of complex conjugate eigenvalues can be treated in the same way as real eigenvalues. That is, the expression for the convolution of eigenvalues yields a proper PDF even when the parameters include pairs of complex conjugate eigenvalues. This is the case as corresponding factors of the exponentials are complex conjugates as well. Now, irrespective of the eigenvalues being real or not, a similar derivation of the delay distribution for systems with forward bypass reactions fails, as additional entries below the sub-diagonal of the rate matrix impede the calculation of its *M*_1,*n*_ minor (a major step in the derivation of the delay PDF in [20]).

In a fully reversible reaction chain, any backward bypass reaction as seen from one end of the linear reaction scheme becomes a forward bypass reaction when seen from the opposite end of the reaction chain, and vice versa. In this case, equation (3.3) yields the first-arrival delay distribution only for the walker for which the bypass is ‘backward’, that is, opposite to the walker's direction. For instance, *S_j_* → *S*_*i*_ with *i* > *j* + 1 is a forward bypass for a walker starting in *S*_1_ and arriving in *S_n_* but a backward bypass for a walker starting in *S_n_* and arriving in *S*_1_. Hence, equation (3.3) yields the first-arrival-at-*S*_1_-delay distribution, but not the first-arrival-at-*S_n_* distribution. Also, in the presence of either forward or backward bypass reactions, the minors *M*_1,1_ and *M_n_*_−2,*n*−2_ in the first-return distributions, namely equations (4.1) and (4.2), are no longer tridiagonal. Hence, they need to be computed with alternative methods. However, if the eigenvalues of rate matrix 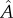 are all simple, equations (4.1) and (4.2) remain correct in the presence of both forward and backward bypass reactions—as long as these do not produce any SOI (see the electronic supplementary material, §S5).

So, equation (3.3) is not correct if and when the system has bypass reactions. In this case, assuming again that the eigenvalues are all simple and that bypass reactions do not produce any SOI, one needs to use5.1
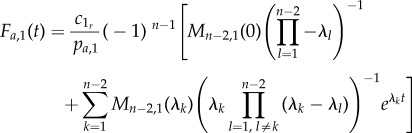
and5.2
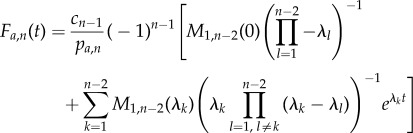
with
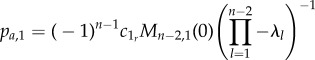
and
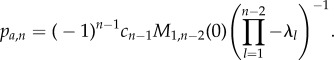


Note that here we recover equation (3.3) from (5.1) and (5.2) for tridiagonal minors. Equation (5.1) has to be used for systems with backward bypass reactions, whereas equation (5.2) has to be used for systems with forward bypass reactions. Both equations follow directly from our derivation in the electronic supplementary material, §§S3 and S5. Furthermore, in the electronic supplementary material, §S5, we outline how closed expressions can be derived in the rather infrequent scenario that the eigenvalues are not simple (i.e. they are no longer unique). The expressions associated with these cases are more complex because of additional terms stemming from the partial fraction expansion for non-simple roots.

Lastly, bypass reactions that involve any SOI as a reactant have to be added to the abridgement scheme, and yield two additional delay reactions: one first-return and one first-arrival distribution. Our approach described in the electronic supplementary material, §S5, is general enough to also cover this scenario and to yield a proper CDF.

## 6. Degradation reactions

Degradation reactions, unless applied to any of the SOIs, can all be lumped into one reaction. The latter can be achieved by including a common absorbing state and all degradation reactions in the transition matrix. Since there are no reactions leaving the absorbing state (i.e. reactions with the absorbing species as a reactant) its corresponding matrix column is zero. Hence, our matrix 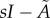 has size (*n* + 1) × (*n* + 1) and its shape is
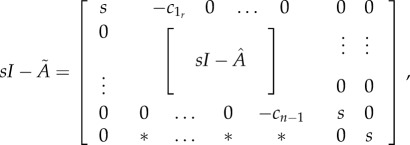
where the symbol ‘
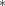
’ in the last row denotes possible individual degradation rates of lumped intermediate species, and the common absorbing state is represented by the last column and row in 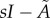. Here, we assume that backward and forward bypass reactions do not produce any SOIs. The determinant of this matrix can then be written as
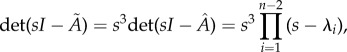
where the *λ_i_*, *i* = 1 … *n* − 2 are the eigenvalues of 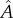, while 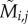, the (*i*,*j*)-minor of 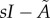, can be written as
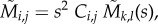
where 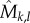 is the (*k*,*l*)-minor of 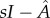 for appropriate *k* and *l* (see the electronic supplementary material, §S5).

Thus, we obtain the same expression for *F*(*s*) as in the electronic supplementary material, §S5, by cancelling out *s*^2^ in the nominator and denominator of the fraction:
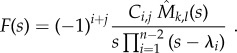


In other words, additional degradation reactions do not lead to changes in the closed expressions for the CDFs of first-return and first-arrival times. Note also that we obtain the probabilities of degradation as *p*_deg,1_ = 1 − *p_r_*_,1_ − *p_a_*_,*n*_ and *p*_deg,*n*_ = 1 − *p_r_*_,*n*_ − *p_a_*_,1_, where the first probability refers to degradation on a walk starting at *S*_1_, and the second to degradation on a walk starting at *S_n_*.

## 7. Synthesis reactions

Synthesis reactions introduce new molecules to the system. Reactions producing SOIs are simply taken over into the abridged system. If a synthesis reaction 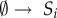 produces one of the intermediate species that are not represented in the abridged scheme, then additional delayed reactions 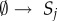 need to be added, namely one for each SOI *S_j_* that the intermediate species can transform into. For example, in the case of the fully linear reaction chain with two SOIs, *S*_1_ and *S_n_*, one would need to add two delayed reactions, 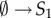 and 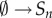, each associated with a specific delay distribution. Such distributions are then obtained as first-arrival-at-*S*_1_ and first-arrival-at-*S_n_* distributions *F_i_*_,1_ and *F_i_*_,n_, respectively. Their derivation follows the steps in the electronic supplementary material, §S5.

## 8. Reduction example I

**Figure 2.**
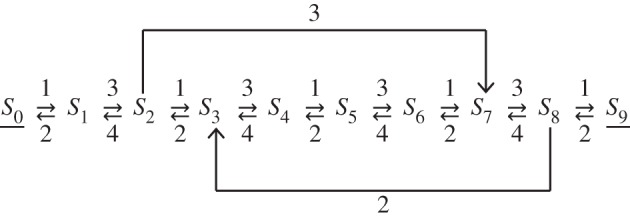
Reaction system consisting of a fully bi-directional chain between the two SOIs *S*_0_ and *S*_9_ and two uni-directional bypass reactions. Rates are noted next to the reaction arrows.

We illustrate the accuracy and efficiency of our approach by exploring the system illustrated in figure 2. Here, we mark the two SOIs (*S*_0_ and *S*_9_) with underbars.

**Figure 3.**
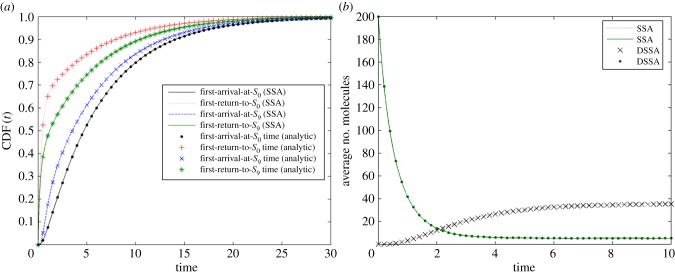
(*a*) First-arrival and first-return delay distributions obtained from the corresponding analytic expressions (dots) and via SSA sampling (solid lines). (*b*) Mean number of SOI molecules (*S*_9_ and *S*_0_) obtained from 1000 simulations of the SSA (solid line) on the original model and the rejection DSSA (dots) on the abridged model, each. The initial conditions are *S*_9_(*t* = 0) = 200 and *S*_0_(*t* = 0) = 0 (S*i*(t = 0) = 0 for *i* = 1 … 8 in the unabridged model).

We adopted the reduction methodology described in §5, and compared with simulations of the full system. Results can be found in figure 3. There, figure 3*a* visually shows the accuracy of our method, with a perfect match between CDFs of all four distributed delays obtained analytically (dots) or by sampling from SSA simulations (solid lines). Figure 3*b* illustrates the dynamics of the system over 10 time units when starting in state (*S*_0_, *S*_9_) = (0, 200). For this example, the speed-up of DSSA (rejection method, [24]) over SSA simulations is about sixfold.

**Figure 4.**
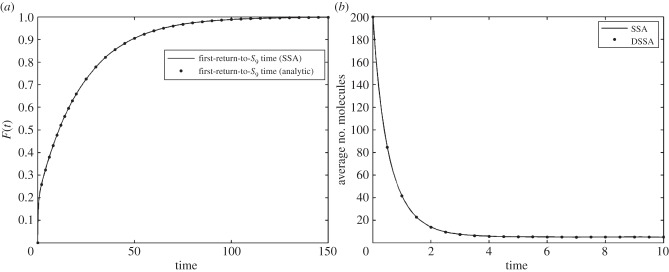
(*a*) First-return delay distribution obtained from the analytic expression (dots) and via SSA sampling (solid line). (*b*) Mean number of SOI *S*_0_ obtained from 1000 simulations of the rejection DSSA of the abridged model. The initial condition is identical to the one noted in the caption to figure 3.

When lumping the same system to just one SOI *S*_9_ with a single delayed reaction *S*_9_ → *S*_9_, we obtain the first-return distribution shown in figure 4*a*. Evidently, the sampled and the analytically derived CDFs of the first-return distribution are identical. Note that the first-return-to-*S*_9_ delay distribution used here is different from the one in figure 3*a*, since the delay now has to also include walks that arrive at *S*_0_. Then, by plugging this distribution into the DSSA, we obtain the same result as above (figure 4*b*) with a 15-fold speed-up over SSA simulations.

It is important to note that initial conditions of a full, original system are in general not readily transferable to corresponding initial conditions of the abridged system. Only the numbers of molecules of SOIs in the latter remain identical to those in the original model. By contrast, the molecules of abridged species all together correspond to a history in the abridged model. That is, such molecules have to be represented by individual delays (one delayed reaction per molecule). Obtaining such delays in turn requires the calculation of additional delay distributions: for each abridged species *S_i_* with at least one initial molecule in the unabridged model, one has to calculate the first-arrival times to any given SOI in the abridged system together with the associated probabilities of arrival. Then, prior to any DSSA simulation, the delay history is constructed by drawing a delayed reaction for each non-SOI molecule. A delayed reaction is chosen by first drawing an arrival state, using the calculated probabilities of arrival, and then drawing the update time point from the corresponding delay distribution, which is determined by the starting species (non-SOI) and the arrival species (SOI). All such drawn delayed reactions will enter the DSSA's update queue for delayed reactions. For details of the DSSA implementation, we refer to [24].

## 9. Reduction example II: glycolysis

Metabolic pathways are composed of interconnected biochemical reactions where, typically, the products of one reaction are the substrates of subsequent reactions. In this section, we will focus on glycolysis [26], one well-known metabolic system. The end result of glycolysis is the breakdown of glucose, but several reactions in this autocatalytic pathway are reversible, contributing to gluconeogenesis. The latter is the generation of glucose, and it is one of the main mechanisms with which several animals regulate blood glucose levels. Importantly, when glucose enters a cell, ATP phosphorylates it irreversibly.

The specific glycolysis example we present here is a minimalist model that was recently shown to preserve some basic dynamic properties [27,28]. The basic components of this glycolysis model [27] are the production of ATP, which is also consumed early in the pathway, and the transcription of phosphofructokinase (PFK), an enzyme that is downregulated by ATP. Besides these two elements, there are a number of intermediate species that represent the inner loop of the glycolysis pathway. It is worth noting that this model does not show the enzymes involved in catalysing the allosteric reactions, and all species can be consumed through a non-specific consumption process.

The reaction scheme of the glycolysis model in [27] has the form
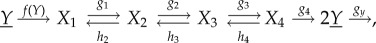
where *Y* denotes ATP. The activity of PFK is only implicitly modelled here as part of the initial autocatalytic reaction, with a rate
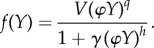


For illustration purposes, we will not consider the degradation of intermediate species *X_i_* at this moment. However, extending the scheme to degradations is easily possible within our abridgement framework.

**Figure 5.**
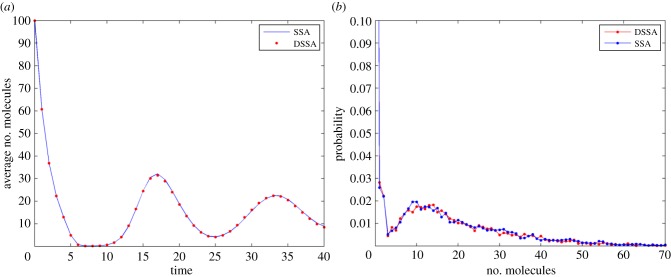
(*a*) Number of *Y* molecules over time for the original system (SSA) and the abridged system (DSSA), each averaged over 10 000 simulations. (*b*) Histograms for the number of *Y* molecules at *t* = 40.

We will use the following abridgement scheme with one delayed and one non-delayed reaction:
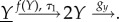


For simulations, the following parameters were selected: *V* = 500, *φ* = 0.5, *q* = 2, *h* = 5, *γ* = 2, *g_i_* = 2 for *i* = 1 … 4, *g_y_* = 0.5, *h_i_* = 1 for *i* = 2 … 4. Our initial condition was set to *Y* = 100. Figure 5*a* shows the average trajectories of both the SSA and DSSA over 10 000 simulations each, and figure 5*b* shows a comparison of histograms of the number of *Y* molecules at time *t* = 40 (arb. units). For such parameter values simulations over 100 time units of the abridged system are about 1.6 times faster than SSA simulations of the original system.

## 10. Reduction example III: kinetic proofreading in T-cell receptor signalling

Upon ligand binding, T-cell receptor complexes undergo a series of modifications before transmitting a signal. In [29], the author developed a kinetic proofreading model that shows how the temporal lag created by intermediate modifications may greatly enhance the ability of the receptor to discriminate foreign antigens from self-antigens with moderately lower affinity.

The underlying processes in T-cell receptor signalling are constituted by a long chain of reactions, activating a ligand–receptor complex via tyrosine phosphorylation and/or recruitment of additional components. However, complex dissociation is also possible, reversing modifications through phosphatases.

The proposed reaction scheme of T-cell kinetic proofreading in [29] is


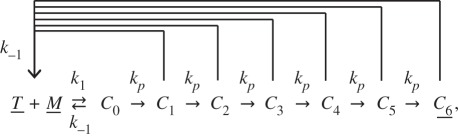


where all SOIs appear underlined. Here, nascent complexes formed from the binding of the T-cell receptor (*T*) and a peptide (*M*) undergo *N* intermediate steps (*N* = 6, which has been adjusted to a rather conservative example in the number of intermediate steps [29]), before generating the active complex, from which major signals are then transmitted.

We will now use the following abridgement scheme with two delayed reactions and one non-delayed reaction:
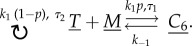


The symbol 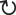 refers to a reaction that consumes its reactants (here, *T* + *M*) and produces the same reactants again after a specified delay (*τ*_2_). *p* is the probability that the final and fully active complex *C*_6_ is produced and, hence, 1 − *p* is the probability that a complex, while undergoing modifications, dissociates.

**Figure 6.**
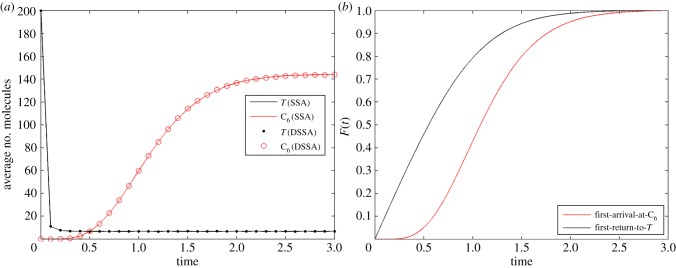
(*a*) Number of molecules of SOIs *T* and *C*_6_ over time for both the original system simulated with the SSA and the abridged system simulated with the DSSA, each averaged over 1000 simulations. (*b*) Delay distributions (CDFs) used for the two delayed reactions in the abridged model.

Figure 6*a* shows simulation results and figure 6*b* shows the two delay distributions obtained for parameters *k*_1_ = 1, *k*_−1_ = 0.25, *k_p_* = 5, with initial conditions *T* = *M* = 200 and *C*_6_ = 0.

For this set of parameters, we did not observe any speed-up (nor any slow-down) between the original and lumped model simulation times. However, additional simulations were performed considering different parameter settings, highlighting trends in computational savings. For instance, when *k*_−1_/*k_p_* ∼ 10^−3^, the abridged model with the DSSA was faster (a speed-up factor of approx. 1.4 for the abridged case). In the opposite case, *k_p_*/*k*_−1_ ∼ 10^−3^, the original model simulated with the SSA was faster (here, a slow-down of approx. 0.4 was observed). As in the examples above, speed-ups are only substantial when the number of SSA reactions is very large, and this highly depends on the choice of parameters.

## 11. Discussion

Simulating biochemical processes accurately can be a painstaking task. Realistically, choosing the correct model is not always straightforward and, even when deriving a suitable representation, solving/simulating such models presents its own hurdles. For instance, networks of chemical interactions can be very large, and involve multiple time scales. Thus, simulations considering large numbers of molecular species in relevant time scales can be computationally expensive, if not unfeasible. As a consequence, a lot of effort has been put into either accelerating existing simulation techniques or obtaining reasonable approximations to real dynamics.

Recently, we presented a novel methodology reducing certain types of chemical systems exactly, while yielding large computational savings, of several orders of magnitude [20]. The latter was achieved by substituting large numbers of chemical reactions with prescribed time delays, outputting an exact solution for a user-defined SOI. Additionally, blocks of lumped reactions could be connected with each other. This provided a clean strategy to break large systems into manageable parts, retaining full accuracy of the selected SOI. However, exactness in the dynamics of the SOI required the irreversibility of the first and last reaction of the chain.

In this paper, we extended our methodology in [20], and provided derivations for all associated delay distributions in analytical, closed form. The latter includes all types of bypass, degradation and synthesis reactions. Additionally, we were able to provide a methodology for fully reversible chains, where the SOI can also be involved in reversible reactions. Each of the possible model extensions was introduced, one by one, and a general formulation for all simultaneous possible extensions can be found in the electronic supplementary material, §S5. The analytical expressions obtained further alleviate computational overheads. Namely, those associated with deriving delay distributions numerically by matrix exponentiation. By consequence, total computational costs can be significantly reduced. A step-by-step summary of the reduction methodology can be found in the electronic supplementary material, §S6.

It is worth noting that the correct definitions of SOIs, first-return times and first-arrival times constitute a crucial step. Our methodology can exactly reduce bi-directional chains of monomolecular reactions, along with parallel bypass, degradations and synthesis reactions. However, modellers may even define more intricate systems. For instance, bimolecular reactions are allowed at the beginning and end of linear reaction chains. Also, two chains of chemical interactions that are connected by a bimolecular reaction can each be lumped by defining reactants involved in the bimolecular reaction as SOIs.

We illustrated some of the computational savings achievable by using our methodology by comparing the computational costs of stochastic simulations of unabridged systems (using the SSA) with those of the corresponding lumped systems with delays (using the DSSA). Additional simulation scenarios can be found in the electronic supplementary material with a larger number of bypasses, separation of time scales between rate parameters, and the consideration of non-SOI initial conditions. We also presented two applications in biology: reduction of a glycolysis model and kinetic proofreading in T-cell signalling.

The applicability of our methodology is not limited to biochemical reaction networks. It extends to Markov-chain models and other processes consisting of interconnected, subsequent transformation steps. For example, in chemistry, the study of polymerization processes such as the formation of soot in hydrocarbon combustion [30] could greatly benefit from our technique. The same holds for oxidation processes, for instance hydrocarbon oxidation [31], as they involve a large number of functional isomers and isomerization reactions.

Depending on the case, the computational savings can be quite significant. However, it should be noted that the SSA is by now standard, and several algorithms have been implemented to make it as efficient as possible [32]. In comparison, only two exact DSSAs exist: the rejection method [24] and the direct DSSA [25]. It would be interesting to see the extent to which full optimization of the DSSA code will yield further computational cost reductions. A combination with available delayed tau-leap methods [21] might also be an interesting direction to explore and is likely to reduce computational costs further. However, as is the case in any coarse-grained method, exactness would no longer be guaranteed, and a compromise between cost and accuracy would be in place.

Lastly, it should be noted that our method is also applicable when modelling chemical reaction networks deterministically. In this case, the set of ODEs describing each species should be replaced by a reduced set of delay differential equations—one for each SOI. We have not carried out any performance studies for deterministic models but it is reasonable to assume that our methodology will gain computational savings also for very large, deterministic systems.
